# *Magnolia officinalis* Extract Contains Potent Inhibitors against PTP1B and Attenuates Hyperglycemia in db/db Mice

**DOI:** 10.1155/2015/139451

**Published:** 2015-05-07

**Authors:** Jing Sun, Yongsen Wang, Xueqi Fu, Yingli Chen, Deli Wang, Wannan Li, Shu Xing, Guodong Li

**Affiliations:** ^1^Edmond H. Fischer Signal Transduction Laboratory, School of Life Sciences, Jilin University, Changchun 130012, China; ^2^Academy of Traditional Chinese Medicine and Chinese Medicinal Materials of Jilin Province, Changchun 130012, China; ^3^Changchun Institute of Applied Chemistry, Chinese Academy of Sciences, Changchun 130022, China; ^4^Department of Colorectal and Anal Surgery, The Second Hospital, Jilin University, Changchun 130041, China

## Abstract

Protein tyrosine phosphatase 1B (PTP1B) is an established therapeutic target for type 2 diabetes mellitus (T2DM) and obesity. The aim of this study was to investigate the inhibitory activity of *Magnolia officinalis* extract (ME) on PTP1B and its anti-T2DM effects. Inhibition assays and inhibition kinetics of ME were performed *in vitro*. 3T3-L1 adipocytes and C2C12 myotubes were stimulated with ME to explore its bioavailability in cell level. The *in vivo* studies were performed on db/db mice to probe its anti-T2DM effects. In the present study, ME inhibited PTP1B in a reversible competitive manner and displayed good selectivity against PTPs *in vitro*. Furthermore, ME enhanced tyrosine phosphorylation levels of cellular proteins, especially the insulin-induced tyrosine phosphorylations of insulin receptor *β*-subunit (IR*β*) and ERK1/2 in a dose-dependent manner in stimulated 3T3-L1 adipocytes and C2C12 myotubes. Meanwhile, ME enhanced insulin-stimulated GLUT4 translocation. More importantly, there was a significant decrease in fasting plasma glucose level of db/db diabetic mice treated orally with 0.5 g/kg ME for 4 weeks. These findings indicated that improvement of insulin sensitivity and hypoglycemic effects of ME may be attributed to the inhibition of PTP1B. Thereby, we pioneered the inhibitory potential of ME targeted on PTP1B as anti-T2DM drug discovery.

## 1. Introduction

Diabetes mellitus (DM) is a chronic disease accompanied by a series of metabolic disorders due to insulin deficiency or impaired insulin action. The prevalence of DM is increasing globally. According to the latest data from the International Diabetes Federation (IDF), 382 million people are living with diabetes in 2013, and this figure will rise up to 592 million by 2035 [1]. Type 2 diabetes mellitus (T2DM) is the most common form, which accounts for around 90% of all diabetes worldwide. It is characterized by hyperglycemia and dyslipidaemia, due to a dysfunction of insulin to activate signaling pathways (insulin resistance) in metabolic target tissues [2, 3]. T2DM may result in severe complications, including renal failure, blindness, slow wound healings, and cardiovascular diseases [4].

Protein tyrosine phosphatases (PTPs), the regulators of tyrosine phosphorylation dependent cellular events, play an important role in numerous critical physiological processes and metabolism [5]. Malfunction of PTPs activity has significant implications in many human diseases [5]. PTP1B is the first member of PTPs superfamily isolated from human placenta and characterized as a ~50 kDa protein (435 amino acid residues), consisting of an N-terminal catalytic domain and a C-terminal segment. PTP1B is a ubiquitously expressed nonreceptor tyrosine phosphatase and contains a highly conserved catalytic motif, in which Cys215 and Arg221 are crucial for its phosphatase catalytic activity [6, 7].

PTP1B is a key negative regulator of insulin signaling pathway, which blocks the insulin-stimulated tyrosine phosphorylation of the IR and thereby the insulin receptor substrate-1 (IRS-1) [8, 9]. PTP1B-null mice displayed enhanced tyrosine phosphorylation of IR and IRS-1 in muscle and liver as a consequence of increased systemic insulin sensitivity. Moreover, these PTP1B-null mice were viable and resistant to T2DM and obesity when fed with a high fat diet [10, 11]. On the basis of these findings, screening inhibitors of PTP1B has provided a definitive strategy in the therapy of T2DM and obesity. Antisense oligonucleotides of PTP1B (depletion of PTP1B expression) and BEOV (bis-ligand oxovanadium (IV) compound, a PTP1B inhibitor) have already entered phase II clinical trials [12, 13].

Magnolia bark is a traditional Chinese medicine, known under the name of* houpu* (*Magnolia officinalis* Rehder & E. H. Wilson). It is also a drug-food resource identified by the Ministry of Health of the People's Republic of China. The principal substantial compounds in magnolia bark are phenolic compounds and terpenoids. Magnolia bark has been used for years to treat a variety of disorders including anxiety, nervous disturbances, “stagnation of qi” (low energy), asthma, and digestive problems [14]. In the present study, we prepared* Magnolia officinalis* extract (ME) from dried root barks of* Magnolia officinalis*. We found that ME contains potent inhibitors of PTP1B and displays high selectivity towards PTPs. Meanwhile, ME can activate insulin signaling pathway in 3T3-L1 adipocytes and C2C12 myotubes. More importantly, it can dramatically lower the blood glucose levels in db/db mice. The data highlighted the inhibitory potential of ME targeted on PTP1B as anti-T2DM drug discovery.

## 2. Materials and Methods

### 2.1. Materials and Chemicals

All the dried magnolia barks were purchased from the Jilin Grand Pharmacy and were identified by Academy of Traditional Chinese Medicine and Chinese Medicinal Materials of Jilin Province, China, as* Magnolia officinalis* Rehder & E. H. Wilson. Recombinant proteins containing the catalytic domains of protein tyrosine phosphatase 1B (PTP1B), T cell protein tyrosine phosphatase (TCPTP), Src homology 2 (SH2) domain-containing tyrosine phosphatase 1 (SHP1), Src homology 2 (SH2) domain-containing tyrosine phosphatase 2 (SHP2), and hematopoietic protein tyrosine phosphatase (HePTP) were purified as previously described [15–18]. Bovine serum albumin (BSA), Tris, p-nitrophenyl phosphate (pNPP), and PVDF membranes were purchased from Milipore. 3T3-L1 preadipocytes and C2C12 myoblasts were obtained from KeyGEN Biotech, and all cell culture reagents were from GIBCO. Antiphosphotyrosine (sc-7020), anti-GLUT1 (sc-7903), anti-GLUT4 (sc-1608), and anti-phospho-ERK1/2 (sc-7383) antibodies were purchased from Santa Cruz. Anti-IR*β* (#3025) and anti-phospho-IR*β* (#3021) were purchased from Cell Signaling Technology. Anti-ERK1/2 (bs-0022R) and HRP-conjugated secondary antibodies were purchased from Beijing Biosynthesis Biotechnology. Anti-*β*-actin (TA-09) was purchased from ZSGB-BIO. The necessary apparatus for SDS-PAGE and Western blot were bought from Bio-Rad.

### 2.2. Preparation of ME

ME was extracted from the dried root barks of* Magnolia officinalis*. Briefly, the dried root barks of* Magnolia officinalis* were extracted three times through condensate reflux with 95% methanol (*W* : *V* = 1 kg : 10 L) in 55°C water bath, then filtered, and concentrated to obtain the methanol extract. The methanol extract was dissolved in distilled water (*W* : *V* = 1 : 1) and extracted three times with n-hexane at a volume ratio of 1 : 1 and aqueous phase I was separated. The aqueous phase I was extracted three times with dichloromethane at a volume ratio of 1 : 1 to obtain aqueous phase II. The aqueous phase II was extracted three times with ethyl acetate at a volume ratio of 1 : 1, and ethyl acetate layer was concentrated with a rotary evaporator in 50°C water bath to obtain ME.

### 2.3. PTP Inhibition Assays

The PTPs activities were measured by addition of 10 *μ*L of 100 mmol/L* p*NPP (as the substrate) into a buffer solution (pH 7.0) containing 50 mM MOPS, 100 mM NaCl, 1 mM ethylene diamine tetra-acetic acid (EDTA), 1 mM DL-dithiothreitol (DTT), 1 mg/mL BSA, and 50 *μ*g/mL recombinant PTP1B or other PTPs, along with or without different concentrations of ME diluted in dimethyl sulfoxide (DMSO). After incubation for 10 min at 37°C, the reactions were terminated with 0.1 M NaHCO_3_, and the amount of the product,* p*-nitrophenol (*p*NP), was measured by UV absorbance at a wavelength of 405 nm. The inhibitory potency of inhibitors was evaluated by IC_50_ values.

### 2.4. Determination of Inhibition Kinetics

Substrate (*p*NPP) with different concentrations was added separately to the reaction mixtures containing various concentrations of ME. The absorbance at 405 nm was measured to determine the amount of the produced* p*-nitrophenol. The inhibiting kinetic analysis was carried out according to the Lineweaver-Burk plot, 1/*v* versus 1/[*S*].

### 2.5. Cell Culture and Differentiation

3T3-L1 preadipocytes and C2C12 myoblasts were cultured in Dulbecco's Modified Eagle's Medium (DMEM), supplemented with 10% fetal bovine serum (FBS) and 1% penicillin/streptomycin (PS) at 37°C in humidified atmosphere with 5% CO_2_. For differentiation, 3T3-L1 preadipocytes were grown to confluence and further cultured for an additional 2 days. Differentiation was initiated by culturing in DMEM with 10% FBS, 1% PS, 0.2 nM insulin, 0.25 *μ*M dexamethasone (DEX), and 0.5 mM 3-isobutyl-1-methylxanthine (IBMX) for 2 days. Then, the medium was changed to DMEM with 10% FBS, 1% PS, 0.2 nM insulin for another 2 days. Finally, the medium was changed to DMEM with 10% FBS and 1% PS for full maturation into adipocytes. The differentiation of 3T3-L1 preadipocytes was detected by oil red O staining [19]. C2C12 myoblasts were stimulated to differentiate into myotubes according to a standard method [20]. Briefly, C2C12 myoblasts were cultured to 75% confluence in DMEM with 10% FBS, 1% PS. Then, cells were cultured in differentiation medium (DMEM supplemented with 2% horse serum and 1% PS) for 4–6 days to mature myotubes.

### 2.6. Western Blot

The tyrosine phosphorylation of cellular proteins was detected in mature 3T3-L1 adipocytes and C2C12 myotubes. Cells were stimulated with ME at various concentrations for 30 min. The effects of ME on insulin signaling were also investigated in both 3T3-L1 adipocytes and C2C12 myotubes. After serum-free starvation for 4 h, cells were incubated with vehicle or ME (dissolved in DMSO) at various concentrations for 30 min and then stimulated with vehicle or 10 nM insulin (dissolved in sterile water with pH 4.0) for 5 min. All the stimulations were stopped by ice-cold PBS. Then the stimulated cells were lysed in ice-cold whole cell extraction buffer (WCEB) containing 25 mM *β*-glycerophosphate (pH 7.3), 5 mM EDTA, 2 mM EGTA, 5 mM *β*-mercaptoethanol, 1% Triton X-100, 0.1 M NaCl, and a protease inhibitor mixture (Roche Applied Science), and the lysates were centrifuged at 12,000 g for 15 min at 4°C. The supernatants were collected individually and the protein concentrations were determined by use of Bradford methods. To investigate the GLUT4 translocation, cells were incubated with ME and then insulin as above. The cell membrane fractions were harvested according to membrane protein extraction kit (BestBio, China). The proteins were separated on a 10% SDS polyacrylamide gel and electrotransferred to polyvinylidene fluoride (PVDF) membrane. Western blot analysis was performed with antibodies against actin, phosphotyrosine, phospho-IR*β*, anti-IR*β*, phospho-ERK1/2, ERK1/2, GLUT1, and GLUT4. Proteins were visualized using the enhanced chemiluminescence (ECL) method and quantified by densitometry scanning with Quantity One software.

### 2.7. Animal Experiments

Twenty SPF grade db/db mice (from the Model Animal Research Center of Nanjing University), male, 8–10 weeks of age, were selected for the studies. The mice were randomly divided into two groups with 10 mice in each group. Group 1: diabetic control mice treated through oral gavage with 0.9% saline (vehicle). Group 2: diabetic mice treated through oral gavage with 0.5 g/kg dose of ME (0.1 mL/10 g body weight) once a day for 4 weeks. All animal trial procedures instituted by the Ethical Committee for the Experimental Use of Animals and for Drug Safety Evaluation in Jilin University were followed. All mice were housed 5 in a cage and maintained at ambient temperature of 22 ± 2°C in a 12:12 h light/dark cycle with free access to food and water. During the treatment period, body weight and blood glucose of 16 h fasted mice were monitored every week.

### 2.8. Acute Toxicity Assay

Acute toxicity of ME was determined by use of Chinese Kunming mice (Mus musculus). All animal trial procedures instituted by the Ethical Committee for the Experimental Use of Animals and for Drug Safety Evaluation in Jilin University were followed. Fifty Chinese Kunming mice of both sexes, 8 weeks, 20–22 g weight (from the Experimental Animal Center of Jilin University), were randomized into five groups with 5 males and 5 females in each group and orally treated with ME once in dose of 3.2, 2.56, 2.048, 1.638, and 1.311 g/kg, respectively. The occurrence of massive toxic reactions and/or death was observed during the first 30 min periodically during the first 24 hours after administration of treatments and then daily for the following 14 days. At the end of the observational period, necropsy was performed on each animal to observe macroscopic reactions in brain, heart, kidneys, liver, spleen, stomach, large and small intestine, and lungs. The acute toxicity of ME was preliminarily evaluated by LD_50_ value (the drug dose leading to 50% lethality).

### 2.9. Statistical Analysis

Data are presented as mean ± SEM. Experiments were performed at least three times. Data were analyzed by Student's *t*-test and one-way ANOVA by use of Graph Pad Prism 5 software. Statistical significance was assigned if *P* < 0.05.

## 3. Results

### 3.1. ME Exhibited Potent PTP1B Inhibitory Activity

**Figure 1:**
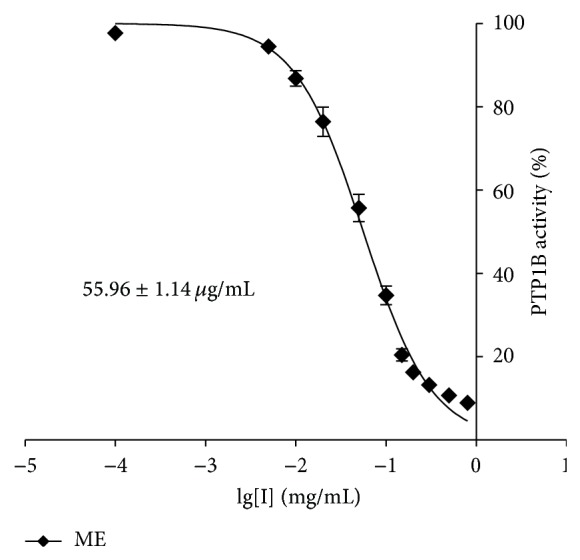
Inhibitory potency of ME. Concentration-dependent inhibitory curve of PTP1B by ME.

The inhibitory potency of ME on the PTP1B was evaluated according to the concentration-dependent inhibition curves as shown in Figure 1. ME was identified as a PTP1B inhibitor, with an IC_50_ of 55.96 *μ*g/mL. Our results also demonstrated selectivity of ME towards PTPs (Table 1). We determined the content of magnolol and honokiol in ME by using HPLC-MS/MS. The content of magnolol and honokiol in ME is about 50% and 5%, respectively (Figure S1 available online in Supplementary Material at http://dx.doi.org/10.1155/2015/139451).

### 3.2. ME Inhibited PTP1B in a Competitive Manner

**Figure 2:**
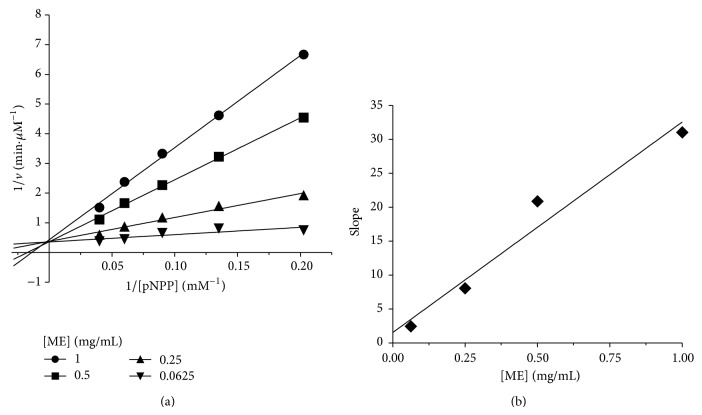
Inhibition kinetic analysis of ME against PTP1B. (a) Lineweaver-Burk plot of 1/*v* (min. *μ*M^−1^) versus 1/[pNPP] (mM^−1^) at various fixed concentrations of ME. (b) The inhibitor constant (Ki) of ME against PTP1B.

In order to further determine the inhibition mode of the ME on PTP1B, Lineweaver-Burk analysis was conducted. As shown in Figure 2, a common intercept of four Lineweaver-Burk lines on the *y*-axis can be obtained as ME concentration increases, suggesting that ME was a typical competitive inhibitor against PTP1B. The inhibitor constant (Ki) was a complex dissociation constant of enzyme with inhibitor. From Figure 2, −1/Ki value was determined from the *x*-axis intercept, and thus Ki was calculated to be 49 *μ*g/mL.

### 3.3. ME Enhanced Tyrosine Phosphorylation of Cellular Proteins

**Figure 3:**
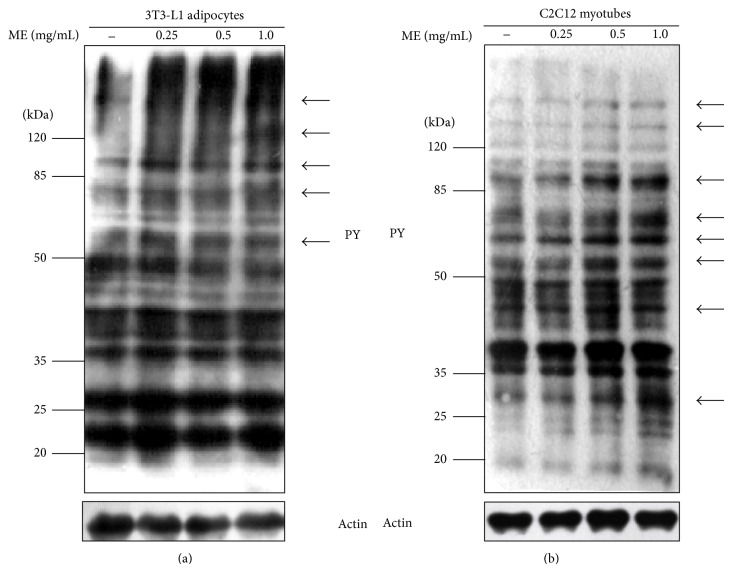
Tyrosine phosphorylation of cellular proteins stimulated by ME. Differentiated 3T3-L1 adipocytes (a) and C2C12 myotubes (b) were stimulated with different doses of ME for 30 min. Equal amounts of total proteins were subjected to Western blot analysis with antibodies against phosphotyrosine (pY) and actin. Arrows indicate proteins whose phosphorylation is significantly enhanced by ME.

According to our knowledge, PTP1B is involved in some critical regulatory steps of signal transduction by dephosphorylation of signaling molecules; therefore, ME presumably increases protein tyrosine phosphorylation by the inhibition of PTP1B. Due to the limitation of the direct method used in detecting PTP1B enzyme activity in cells, we investigated tyrosine phosphorylation of cellular proteins to confirm whether ME has the inhibitory effect on PTP1B. Western blot analysis showed that ME enhances tyrosine phosphorylation of cellular proteins in a dose-dependent manner in 3T3-L1 adipocytes (Figure 3(a)). To confirm our results found in 3T3-L1 adipocytes, we performed the experiment in C2C12 myotubes. As indicated, the tyrosine phosphorylation of cellular proteins has been largely increased in the ME-treated C2C12 myotubes (Figure 3(b)). Therefore, it is acceptable to draw the conclusion that the inhibition of PTP1B is at least partly accounted for by the enhanced tyrosine phosphorylation.

### 3.4. ME Enhanced Insulin Signaling Pathway

Considering that PTP1B directly dephosphorylates IR and IRS-1, thereby negatively regulating insulin signaling pathway, we further explored whether ME could affect insulin signaling pathway.

**Figure 4:**
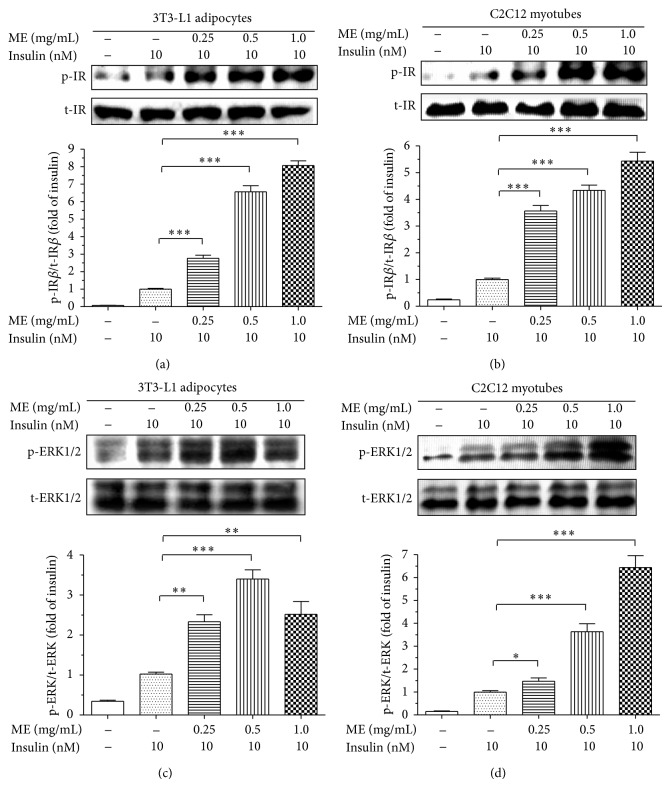
Effects of ME on insulin signaling pathway. Differentiated 3T3-L1 adipocytes (a and c) and C2C12 myotubes (b and d) were starved for 4 h before stimulation. The cells were incubated with vehicle or ME at various concentrations for 30 min and then stimulated with vehicle or 10 nM insulin for 5 min. Tyrosine phosphorylations of IR and ERK were determined by Western blotting with anti-phospho-IR*β* and anti-phospho-ERK1/2 antibodies and were normalized with IR and ERK protein, respectively, which were then calculated as fold changes of insulin alone. Data are presented as mean ± SEM (*n* = 3). ^∗^ *P* < 0.05, ^∗∗^ *P* < 0.01, ^∗∗∗^ *P* < 0.001 versus insulin alone.

Our study indicated that, in 3T3-L1 adipocytes, ME enhanced the insulin-induced tyrosine phosphorylation levels of IR*β* without altering the total protein levels of IR*β* in a dose-dependent manner (Figure 4(a)). These results were confirmed in C2C12 myotubes. As indicated, the insulin-stimulated phosphorylation levels of IR*β* were obviously increased in the ME-treated C2C12 myotubes (Figure 4(b)). Based on the findings that ME sensitizes insulin signaling, we next detected the effects of ME on downstream insulin signaling pathway (ERK pathway). As shown in Figures 4(c) and 4(d), ME also enhanced insulin-induced the phosphorylation levels of ERK in a dose-dependent manner in 3T3-L1 adipocytes, as well as in C2C12 myotubes. We also analyzed the effects of ME on Akt in 3T3-L1 adipocytes and C2C12 myotubes, with similar results to that of IR*β* and ERK1/2 (Figure S2). Moreover, we found that ME alone cannot activate insulin pathway in the absence of insulin (Figure S3). Our findings suggested that improvement of insulin sensitivity and activation of the downstream signaling pathway by ME may be mainly attributed to the inhibition of PTP1B.

### 3.5. ME Promoted GLUT4 Translocation

**Figure 5:**
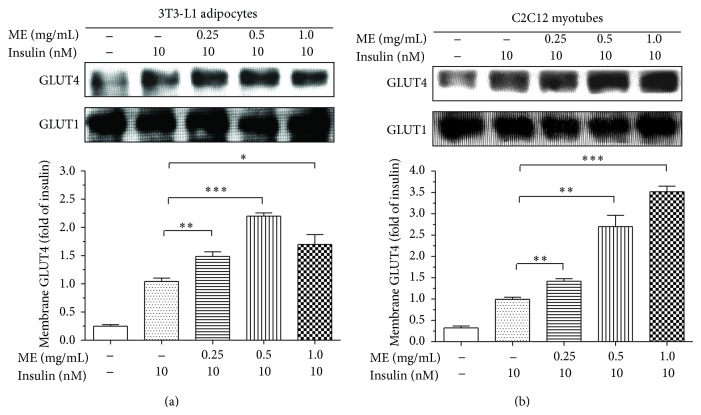
Effects of ME on GLUT4 translocation. Differentiated 3T3-L1 adipocytes (a) and C2C12 myotubes (b) were starved for 4 h before experiment. The cells were incubated with vehicle or ME at various concentrations for 30 min and then stimulated with vehicle or 10 nM insulin for 5 min. The cell membrane fractions were harvested. GLUT4 and GLUT1 were determined by Western blotting with anti-GLUT4 and anti-GLUT1 antibodies. Quantities of GLUT4 in cell membrane fractions were normalized against GLUT1 content and then calculated as fold changes of the insulin alone. Data are presented as mean ± SEM (*n* = 3). ^∗^ *P* < 0.05, ^∗∗^ *P* < 0.01, ^∗∗∗^ *P* < 0.001 versus insulin alone.

Another downstream consequence of insulin signaling is GLUT4 cell membrane translocation. Upon the demonstration that ME can activate insulin-induced ERK pathway, we investigated whether ME is also able to promote GLUT4 translocation. Compared to untreated cells, after stimulation of insulin or insulin plus various concentrations of ME, GLUT4 were aggregated at the cell membrane fractions (Figure 5(a)). The similar results were confirmed in C2C12 myotubes (Figure 5(b)). These results suggested that ME has a potential hypoglycemic function* in vivo*.

### 3.6. ME Decreased Blood Glucose Level in db/db Mice

**Figure 6:**
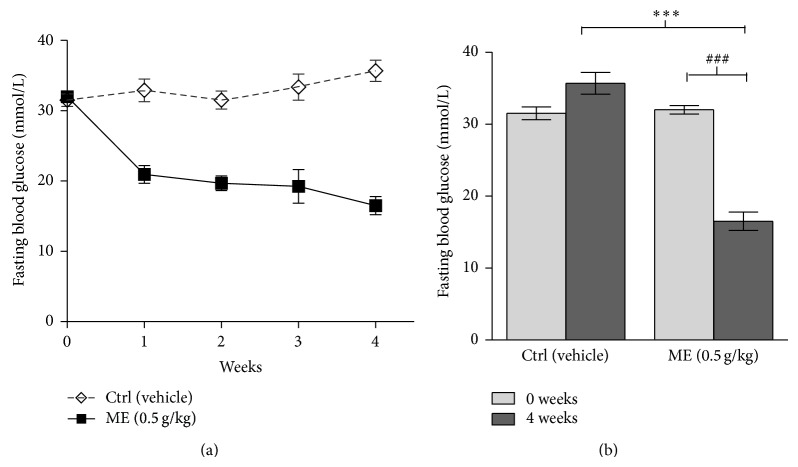
The effects of lowering the blood glucose of ME in db/db mice. Mice were orally treated with 0.9% saline alone (vehicle) and ME (0.5 g/kg) once a day for 4 weeks. Data are presented as mean ± SEM (*n* = 10). ^###^ *P* < 0.001 versus ME group (0 week), ^∗∗∗^ *P* < 0.001 versus diabetic control group (4 weeks).

After the confirmation of the finding that ME enhanced insulin signaling pathway and promoted GLUT4 translocation in cells, we went on to challenge these promising findings with* in vivo* situation. The db/db mice with the genetic mutation of leptin receptors suffer insulin resistance and severe hyperglycemia and thus have been widely used as a model of T2DM. As shown in Figure 6, the blood glucose levels were dramatically decreased after treatment with ME at 0.5 g/kg daily for 4 weeks in db/db mice compared to control group. The result fits well with* in vitro* and cell experiments outlining ME with its bioavailability as an efficient blood glucose lowering agent, though further* in vivo* experiments, particularly with lower administration doses and treatment time, are required to better estimate the therapeutic potential of ME. We also found that the weight gain of db/db mice during the period of intragastric administration with ME can be controlled effectively (Figure S4).

### 3.7. Acute Toxicity Trials of ME

To explore the potential toxicity, we treated Chinese Kunming mice with different dose of ME (Table 2). Lethality of mice in different groups orally treated with ME in dose of 3.200, 2.560, 2.048, 1.638, and 1.311 g/kg was 100%, 80%, 50%, 30%, and 0%, respectively. The occurrences of death were observed in 6 h after administration of treatments. Two mice died in the following day, while the surviving mice were viable over the following 14 days. Thus, the acute toxicity of ME is preliminarily evaluated as median lethal dose with 95% confidence (LD_50_ = 2.0 ± 0.2 g/kg) calculated according to the improved Bliss method. The necropsy was performed on each experimental mouse to observe macroscopic reactions in brain, heart, kidneys, liver, spleen, stomach, intestine, and lungs, and the results showed no lesions. These results indicated that ME has little toxicity and is well tolerated.

## 4. Discussion

PTP1B has been identified as the main negative regulator of insulin signaling. Normally, insulin evokes a cascade of phosphorylation events, starting with the autophosphorylation of IR on multiple tyrosyl residues, which enhances IR kinase activity and leads to recruitment of IRS-1, followed by activation of phosphatidylinositol 3-kinase (PI3K), protein kinase B (PKB; also known as AKT), and finally glucose transporter 4 (GLUT4). Activated GLUT4 could translocate to membrane and thus improve the glucose uptake [21, 22]. Current evidence indicates that PTP1B could dephosphorylate IR with substrate specificity, inhibiting its kinase activity, and thus block insulin signaling transduction [23, 24]. Mice lacking PTP1B gene display improved insulin sensitivity with increased or prolonged tyrosine phosphorylations of IR and stay away from T2DM or obesity [10, 11]. PTP1B knockout mice do not have apparent disease phenotype and keep normal fetal survival rate compared with wild-type controls [10, 11]. Therefore, advisably inhibiting PTP1B activity is considered to evoke multiple series of physiological responses to resist T2DM and obesity via facilitating tyrosine phosphorylation of insulin signaling molecules. In fact, several studies have reported that oral administration of PTP1B inhibitor can decrease glucose in T2DM mice [25–28]. Antisense oligonucleotides of PTP1B (depletion of PTP1B expression) and BEOV (bis-ligand oxovanadium (IV) compound, a PTP1B inhibitor) have already entered phase II clinical trials [12, 13]. These studies indicate that PTP1B inhibitors may be promising candidates for novel antidiabetic drug development. In the present study, we revealed that ME potently inhibits PTP1B* in vitro* and greatly facilitates GLUT4 translocation from cytoplasm to cell membrane by activating insulin signaling pathway in both 3T3-L1 adipocytes and C2C12 myotubes. More importantly, we found that ME can lower blood glucose in db/db mice. Herein, we highlight ME's valuable potential in the development of drug against T2DM.

Although there has been growing concern on developing inhibitors of PTP1B, most of these efforts are in vain due to the challenges arising from the highly structural conservation of PTPs family and toxicity of inhibitor itself. PTP1B shares about 75% sequence identity in its core catalytic domain with TCPTP that is essential for normal hematopoiesis. TCPTP-null mice die as a result of hematopoietic defects within weeks of birth [29]. On the other hand, under physiological conditions, PTP1B modulates insulin signaling pathway in a reversible feedback manner, which warrants the orderly amplification and attenuation of insulin signal. However, most of the noncompetitive inhibitors act via oxidation of the catalytic Cys215 or by preventing the closure of the WPD loop, which are irreversible, consequently bringing toxicity* in vivo*. Our results showed that ME exhibits potent inhibition against PTP1B in a competitive manner and good selectivity towards PTPs* in vitro*. Meanwhile, oral LD_50_ value of ME was 2.0 ± 0.2 g/kg, suggesting the toxicity of ME is at low levels, which may be attributed to its inhibition type and selectivity. In addition, ME enhanced tyrosine phosphorylation of cellular proteins, indicating ME conquers its cell permeability and bioavailability restrictions.

Insulin signaling diverges mainly to the MAPK cascade, leading to the activation of gene expression and growth regulation, and the PI3K-AKT/PKB pathway which further regulates several different metabolic activities including glycogen synthesis and glucose uptake, as well as gene expression and survival [30]. The Western blot results revealed that ME not only augments the phosphorylation of ERK involved in the MAPK pathway though effects of ME on MAPK cascade should be further clarified in future studies, but also promotes GLUT4 translocation which is more relevant to the glucose lowering effects involved in the PI3K-AKT/PKB metabolic pathway. Thus the activation of insulin pathway by ME may be mainly attributed to its potent inhibition against PTP1B. However, considering ME contains a variety of ingredients and signal transduction is a complicated process, the hypoglycemic effect of ME via other mechanisms cannot be fully excluded.

As published, the antihyperglycemic effect of metformin resulted from the suppression of lipid oxidation and hepatic glucose production through AMPK [31]. Our findings verified that ME enhance insulin-stimulated tyrosine phosphorylation of IR*β*, and this effect was transduced downstream to yield a sustained increase in ERK phosphorylation and GLUT4 translocation, indicating that the hypoglycemic mechanism of ME is different from metformin. Interestingly, we also found that ME alone cannot activate insulin pathway (Figure S3). Actually, in the absence of insulin, the insulin pathway maintains a low level in cultured cells. Haque et al. reported that insulin is a protein hormone. It is hard to find some substance insulin-mimetic for its substitution [32]. Therefore, the current evidence revealed that ME functions as an insulin-sensitizer rather than an insulin-mimetic agent.

Magnolia bark is a highly aromatic herbal material from the cortex of* Magnolia officinalis* (Magnoliaceae family). As reported, magnolia bark exerts numerous and diverse pharmacological effects, including anti-inflammatory, antioxidant, antimicrobial, anxiolytic, and antiarrhythmic [14]. The principal substantial compounds in magnolia bark are different phenolic compounds and terpenoids. Several compounds had been identified, including gallic acid, sennosides A and B, hesperidin, naringin, syringin, and especially two neolignan compounds, magnolol and honokiol [14]. Previous report from Sohn et al. revealed magnolol is able to decrease the fasting blood glucose levels in GK rats and attributed the improvement of insulin resistance to chronic glycemic control [33]. In addition, Honokiol has been proved to be a partial nonadipogenic PPAR*γ* agonist* in vitro* which prevented hyperglycemia and weight gain in diabetic KKAy mice [34]. As is well-accepted, potential antidiabetic agents may act via different mechanisms. These significant findings shifted our research object to the magnolia bark, as the initial phase in series of related researches, with an attempt to reveal the inhibitory activity of ME against PTP1B and its potential antihyperglycemic mechanism due to the convenient purchase of magnolia bark from the drug store at a cheap price and easy preparation of ME in the laboratory. Through the experiment results, we are convinced of the effective inhibition of ME against PTP1B. Moreover, our results showed that the magnolol and honokiol are the major components in ME (Figure S1). Based on these findings, further studies on magnolol and honokiol have been carried out in the subsequent experiments to explore their inhibitory activity against PTP1B.

The db/db mice, generated by genetic mutation of leptin receptors resulting in overeating and subsequently obesity and diabetes, have been widely used as a model of T2DM. Here we demonstrated that oral treatment with ME resulted in a profound attenuation in hyperglycemia in db/db mice. The mechanism may be associated with improvement of insulin sensitivity by ME but more studies were needed. It should be mentioned that the db/db model may not fully replicate T2DM in human, although copy number variation at the leptin receptor gene locus is associated with metabolic traits and the risk of T2DM in human subjects [35]. The etiology of T2DM in human may be comprised of genetic and environmental factors. Therefore, further studies are required to validate antihyperglycemic effect of ME in other models of insulin resistance or obesity (e.g., the diet-induced obese model). In addition, oral LD_50_ value of ME indicated that ME is of low toxicity. Hence, ME is promising to benefit T2DM patients without adverse effects although its safety and efficient dosage remain to be validated. Our future research concern goes to the detection of the ME potentials on insulin signaling cascade in muscle, liver, and adipose tissue, and the effects on blood lipid profile (e.g., total cholesterol, LDL-cholesterol, HDL-cholesterol, triglyceride, and free fatty acids), along with the assessment of the cardiovascular effects of ME.

In conclusion, we demonstrated that ME contains potent inhibitors against PTP1B and displays good selectivity towards PTPs* in vitro*; meanwhile ME can efficiently enhance insulin-induced tyrosine phosphorylations of IR*β* in cell level. Furthermore, this effect is transduced downstream to yield a sustained increase in ERK phosphorylation and GLUT4 translocation, which provides a possible mechanism for its antihyperglycemic effects.

## Supplementary Material

The Supplementary Materials involve 4 figures of the quantitative analysis of magnolol and honokiol in ME by HPLC-MS/MS，Effects of ME on Akt，Effect of ME alone on IR, and The effect of ME on body weight of mice, thanks to the reviewer's constructive suggestion to further illustrate the components and functions of ME.

## Figures and Tables

**Table 1:** Selectivity of ME towards a panel of PTPs.

PTPs	IC_50_ (*μ*g/mL) ME
PTP1B	55.96 ± 1.14
SHP1	501.94 ± 11.55
SHP2	521.56 ± 15.76
TCPTP	395.59 ± 9.76
HePTP	677.88 ± 13.25

IC_50_ values of ME against PTPs including PTP1B, SHP1, SHP2, TCPTP, and HePTP were calculated. Data were expressed as mean ± standard error (*n* = 3).

**Table 2:** Acute toxicity trials of ME (*n* = 10).

Groups (ME g/kg)	Deaths	Lethality
3.200	10	100%
2.560	8	80%
2.048	5	50%
1.638	3	30%
1.311	0	0%

## Abbreviations

DM: Diabetes mellitus
T2DM: Type 2 diabetes mellitus
PTPs: Protein tyrosine phosphatases
PTP1B: Protein tyrosine phosphatase 1B
IR*β*: Insulin receptor *β*-subunit
ERK1/2: Extracellular signal-regulated kinases 1/2
GLUT4: Glucose transporter 4
ME: * Magnolia officinalis* extract
IRS-1: Insulin receptor substrate-1
TCPTP: T cell protein tyrosine phosphatase
SHP1: Src homology 2 (SH2) domain-containing tyrosine phosphatase 1
SHP2: Src homology 2 (SH2) domain-containing tyrosine phosphatase 2
HePTP: Hematopoietic protein tyrosine phosphatase
GLUT1: Glucose transporter 1
PI3K: Phosphatidylinositol 3-kinase
PKB: Protein kinase B (also known as AKT)
AMPK: AMP-activated protein kinase.

## Highlights

ME potently inhibits PTP1B* in vitro* and greatly facilitates GLUT4 translocation from cytoplasm to cell membrane by activating insulin signaling pathway in both 3T3-L1 adipocytes and C2C12 myotubes. More importantly, ME can lower blood glucose in db/db mice.

## References

[1] International Diabetes Federation (2013). *IDF Diabetes Atlas*.

[2] World Health Organization (2014). *10 Facts about Diabetes*.

[3] Goldstein B. J. (2002). Protein-tyrosine phosphatases: emerging targets for therapeutic intervention in type 2 diabetes and related states of insulin resistance. *The Journal of Clinical Endocrinology and Metabolism*.

[4] Lin Y., Sun Z. J. (2011). Thyroid hormone potentiates insulin signaling and attenuates hyperglycemia and insulin resistance in a mouse model of type 2 diabetes. *British Journal of Pharmacology*.

[5] He Y., Zeng L.-F., Yu Z.-H., He R., Liu S., Zhang Z.-Y. (2012). Bicyclic benzofuran and indole-based salicylic acids as protein tyrosine phosphatase inhibitors. *Bioorganic & Medicinal Chemistry*.

[6] Tonks N. K., Diltz C. D., Fischer E. H. (1988). Purification of the major protein-tyrosine-phosphatases of human placenta. *Journal of Biological Chemistry*.

[7] Tonks N. K. (2003). PTP1B: from the sidelines to the front lines!. *FEBS Letters*.

[8] Echwald S. M., Bach H., Vestergaard H. (2002). A P387L variant in protein tyrosine phosphatase-1B (PTP-1B) is associated with type 2 diabetes and impaired serine phosphorylation of PTP-1B in vitro. *Diabetes*.

[9] Goldstein B. J., Bittner-Kowalczyk A., White M. F., Harbeck M. (2000). Tyrosine dephosphorylation and deactivation of insulin receptor substrate-1 by protein-tyrosine phosphatase 1B. Possible facilitation by the formation of a ternary complex with the GRB2 adaptor protein. *The Journal of Biological Chemistry*.

[10] Elchebly M., Payette P., Michaliszyn E. (1999). Increased insulin sensitivity and obesity resistance in mice lacking the protein tyrosine phosphatase-1B gene. *Science*.

[11] Klaman L. D., Boss O., Peroni O. D. (2000). Increased energy expenditure, decreased adiposity, and tissue-specific insulin sensitivity in protein-tyrosine phosphatase 1B-deficient mice. *Molecular and Cellular Biology*.

[12] Thompson K. H., Lichter J., LeBel C., Scaife M. C., McNeill J. H., Orvig C. (2009). Vanadium treatment of type 2 diabetes: a view to the future. *Journal of Inorganic Biochemistry*.

[13] Liu G. (2004). Technology evaluation: ISIS-113715, Isis. *Current Opinion in Molecular Therapeutics*.

[14] Jiří P., Jiří J., Anna S. (2006). Expectations of biologically active compounds of the genus Magnolia in biomedicine. *Journal of Applied Biomedicine*.

[15] Liang X., Meng W., Niu T., Zhao Z., Zhou G. W. (1997). Expression, purification, and crystallization of the catalytic domain of protein tyrosine phosphatase SHP-1. *Journal of Structural Biology*.

[16] Shi D., Dong H., Zhu Z. (2007). Expression of catalytic domain of protein tyrosine phosphatase 1B and preparation of its polyclonal antibody. *Chemical Research in Chinese Universities*.

[17] Zhao Z., Bouchard P., Diltz C. D., Shen S.-H., Fischer E. H. (1993). Purification and characterization of a protein tyrosine phosphatase containing SH2 domains. *The Journal of Biological Chemistry*.

[18] Zhu Z.-C., Sun M., Zhang X.-Y. (2007). Expression and characterization of catalytic domain of T cell protein tyrosine phosphatase (Delta TC-PTP)—immunohistochemical study of Delta TC-PTP expression in non-small cell lung carcinomas. *Chemical Research in Chinese Universities*.

[19] Matsuo K., Bettaieb A., Nagata N., Matsuo I., Keilhack H., Haj F. G. (2011). Regulation of brown fat adipogenesis by protein tyrosine phosphatase 1B. *PLoS ONE*.

[20] Shirali S., Bathaie S. Z., Nakhjavani M. (2013). Effect of crocin on the insulin resistance and lipid profile of streptozotocin-induced diabetic rats. *Phytotherapy Research*.

[21] Bryant N. J., Govers R., James D. E. (2002). Regulated transport of the glucose transporter GLUT4. *Nature Reviews Molecular Cell Biology*.

[22] White M. F., Kahn C. R. (1994). The insulin signaling system. *Journal of Biological Chemistry*.

[23] Kenner K. A., Anyanwu E., Olefsky J. M., Kusari J. (1996). Protein-tyrosine phosphatase 1B is a negative regulator of insulin- and insulin-like growth factor-I-stimulated signaling. *The Journal of Biological Chemistry*.

[24] Yip S. C., Saha S., Chernoff J. (2010). PTP1B: a double agent in metabolism and oncogenesis. *Trends in Biochemical Sciences*.

[25] Maeda A., Kai K., Ishii M., Ishii T., Akagawa M. (2014). Safranal, a novel protein tyrosine phosphatase 1B inhibitor, activates insulin signaling in C2C12 myotubes and improves glucose tolerance in diabetic *KK-A^y^* mice. *Molecular Nutrition & Food Research*.

[26] Fukuda S., Ohta T., Sakata S. (2010). Pharmacological profiles of a novel protein tyrosine phosphatase 1B inhibitor, JTT-551. *Diabetes, Obesity and Metabolism*.

[27] Wang Z. Q., Ribnicky D., Zhang X. H. (2011). An extract of *Artemisia dracunculus* L. enhances insulin receptor signaling and modulates gene expression in skeletal muscle in KK-A^y^ mice. *Journal of Nutritional Biochemistry*.

[28] Wang C.-D., Teng B.-S., He Y.-M. (2012). Effect of a novel proteoglycan PTP1B inhibitor from *Ganoderma lucidum* on the amelioration of hyperglycaemia and dyslipidaemia in db/db mice. *British Journal of Nutrition*.

[29] You-Ten K. E., Muise E. S., Itié A. (1997). Impaired bone marrow microenvironment and immune function in T cell protein tyrosine phosphatase-deficient mice. *Journal of Experimental Medicine*.

[30] Taniguchi C. M., Emanuelli B., Kahn C. R. (2006). Critical nodes in signalling pathways: insights into insulin action. *Nature Reviews Molecular Cell Biology*.

[31] Hundal R. S., Krssak M., Dufour S. (2000). Mechanism by which metformin reduces glucose production in type 2 diabetes. *Diabetes*.

[32] Haque A., Andersen J. N., Salmeen A., Barford D., Tonks N. K. (2011). Conformation-sensing antibodies stabilize the oxidized form of PTP1B and inhibit its phosphatase activity. *Cell*.

[33] Sohn E. J., Kim C.-S., Kim Y. S. (2007). Effects of magnolol (5,5′-diallyl-2,2′-dihydroxybiphenyl) on diabetic nephropathy in type 2 diabetic Goto-Kakizaki rats. *Life Sciences*.

[34] Atanasov A. G., Wang J. N., Gu S. P. (2013). Honokiol: a non-adipogenic PPAR*γ* agonist from nature. *Biochimica et Biophysica Acta—General Subjects*.

[35] Jeon J.-P., Shim S.-M., Nam H.-Y. (2010). Copy number variation at leptin receptor gene locus associated with metabolic traits and the risk of type 2 diabetes mellitus. *BMC Genomics*.

