# Supplementary material for: Magnolia officinalis Extract Contains Potent Inhibitors against PTP1B and Attenuates Hyperglycemia in db/db Mice
Source: Biomed Res Int. 2015 May 7;2015:139451. doi: 10.1155/2015/139451 (PMC4439476; doi:10.1155/2015/139451)
Supplement: Supplementary file 1 — The Supplementary Materials involve 4 figures of the quantitative analysis of magnolol and honokiol in ME by HPLC-MS/MS，Effects of ME on Akt，Effect of ME alone on IR, and The effect of ME on body weight of mice, thanks to the reviewer's constructive suggestion to further illustrate the components and functions of ME. [file 139451.f1.pdf]

Supplementary materials

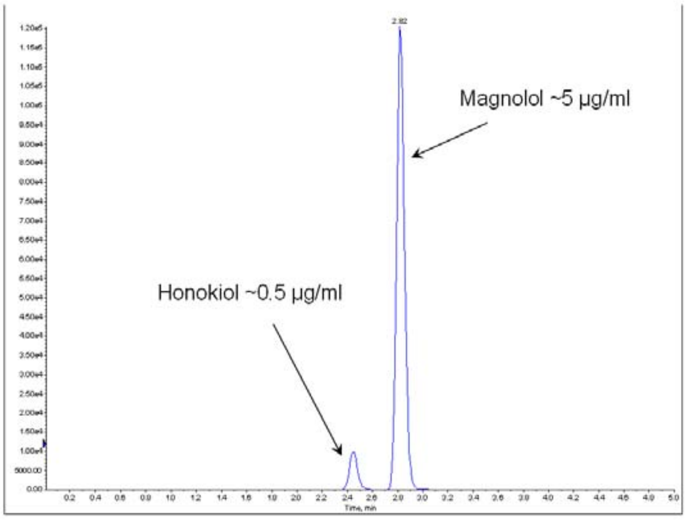

FIGURE S1 The quantitative analysis of magnolol and honokiol in ME by HPLC-MS/MS. The concentration of ME is 10 µg/mL. The content of magnolol and honokiol in ME is about 5 µg/mL and 0.5 µg/mL respectively.

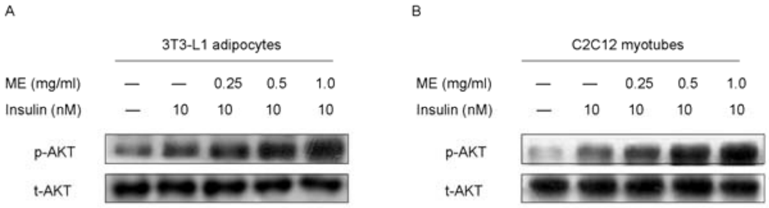

Figure S2 Effects of ME on Akt. Differentiated 3T3-L1 adipocytes (A) and C2C12 myotubes (B) were starved for 4 h before stimulation. The cells were incubated with vehicle or ME at various concentrations for 30 min and then stimulated with vehicle or 10 nM insulin for 5 min. The levels of t-Akt and p-Akt were determined by western blotting with anti-Akt (Cell Signaling Technology #9272) and anti-phospho-Akt (Cell Signaling Technology #9611) antibodies.

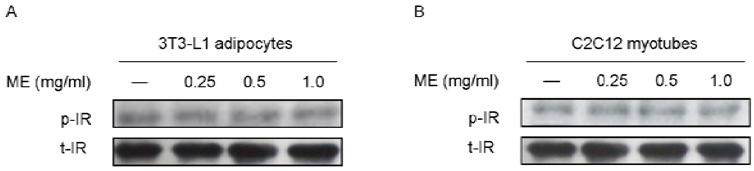

Figure S3 The effect of ME alone on IR. Differentiated 3T3-L1 adipocytes (A) and C2C12 myotubes (B) were starved for 4 h before stimulation. The cells were incubated with vehicle or ME at various concentrations for 30 min. Tyrosine phosphorylations of IR were determined by western blotting with anti-phospho-IRβ antibodies.

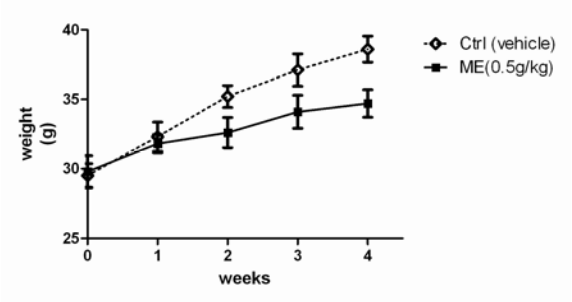

Figure S4 The effect of ME on body weight. Mice were orally treated with 0.9% saline alone (vehicle) and ME (0.5 g/kg) once a day for 4 weeks. Body weight of 16 h fasted mice were monitored every week (n=10).
